# The Treatment of Intussusception

**Published:** 1901-09-07

**Authors:** 


### THE TREATMENT OF INTUSSUSCEPTION.

Speaking at Cheltenham on the subject of intussusception, Mr. D'Arcy Power pointed out in the first instance the rarity of its occurrence, only 65 cases having been met with in the course of 10 years amongst 46,197 surgical cases admitted into the two hospitals with which he is connected. Indeed, he said the Victoria Hospital, which is entirely devoted to the reception of children, only received 15 cases out of 4,164 admissions to the surgical wards. From this it is clear that the experience of no one surgeon on this matter can be very great. Hence the necessity for their comparing notes as to methods and results. Another point was the great fatality of the disease. Forty-two deaths to 23 recoveries was by no means satisfactory, and coming to the consi deration of the treatment of the condition, Mr. D'Arcy Power strongly urged the desirability of early operation. He said that he used to think that it was wise to try to reduce early intussusception by irrigation with hot saline solution, passed into the rectum under a low head of pressure, but a larger experience had taught him that this method was unreliable, and he had become a whole-hearted advocate of abdominal section at the earliest possible moment in every case of intussusception. He related cases which showed, he thought, the danger of treating recurrent intussusception by irrigation or inflation, and the impossibility of telling beforehand whether the condition would or would not recur. So long as the bowel remained invaginated, even to the slightest extent, recurrence was likely to take place. Abdominal section therefore was the proper and only effectual method of treatment, for when perfect reduction had been made there was no further recurrence. Irrigation might still be made use of to mitigate the sufferings of the patient until the services of a surgeon accustomed to abdominal work could be obtained, or might be used by the surgeon himself to reduce the size of the tumour immediately before he operated, but he was convinced that this method of irrigation should not be used to the exclusion of abdominal section, and that medical men should urge parents to allow this to be done at the earliest possible opportunity in every case of intussusception. He further showed that with the rarest exceptions, recovery from the condition, even with operation, only takes place when the invagination is capable of easy and complete reduction. It is the completeness of the reduction which is the key to the whole business, and judging from the remarks made by Mr. Power, it is probably the incompleteness of the reduction, the fact that the last bit is not thoroughly unfolded, that makes the condition recur in so many instances in which after inflation, injection, or other means, relief seems to be, and is for a time, obtained.

